# Umbilical Cord Mesenchymal Stem Cell Transplantation Prevents Chemotherapy-Induced Ovarian Failure via the NGF/TrkA Pathway in Rats

**DOI:** 10.1155/2019/6539294

**Published:** 2019-05-23

**Authors:** Qun Zheng, Xiaoyan Fu, Jinzhan Jiang, Ning Zhang, Libo Zou, Wenqian Wang, Mingxing Ding, Haohao Chen

**Affiliations:** ^1^Center of Clinical Reproductive Medicine, Jinhua People's Hospital, Jinhua, Zhejiang Province, China; ^2^Medical Molecular Biology Laboratory, Medical College, Jinhua Polytechnic, Jinhua, Zhejiang Province, China

## Abstract

Chemotherapy leads to a loss of fertility and reproductive endocrine function, thereby increasing the risk of premature ovarian failure (POF). Studies have suggested that the transplantation of mesenchymal stem cells could inhibit apoptosis in ovarian granulosa cells and improve follicular development. In the present study, the effects of human umbilical cord mesenchymal stem cell (UCMSC) transplantation on ovarian function after ovarian damage caused by chemotherapy and the mechanism underlying these effects were investigated. POF model rats were obtained by the intraperitoneal injection of cyclophosphamide, and cultured UCMSCs were transplanted by tail vein injection. Serum estrogen, follicle-stimulating hormone, gonadotropin releasing hormone, and anti-Mullerian hormone levels were detected by ELISA. Folliculogenesis was evaluated by histopathological examination. The expression levels of nerve growth factor (NGF), high affinity nerve growth factor receptor (TrkA), follicle-stimulating hormone receptor (FSHR), and caspase-3 were evaluated by western blotting and RT-qPCR. The natural reproductive capacity was assessed by pregnant rate and numbers of embryos. The results indicated that UCMSC transplantation recovered disturbed hormone secretion and folliculogenesis in POF rats. NGF and TrkA levels increased, while FSHR and caspase-3 decreased. The pregnancy rate of POF rats was improved. Therefore, UCMSCs could reduce ovarian failure due to premature senescence caused by chemotherapy, and the NGF/TrkA signaling pathway was involved in the amelioration of POF.

## 1. Introduction

Approximately 1.69 million women are diagnosed with cancer each year in China. For the top 5 cancers, 2.78% of patients are under 30 years old and 12.25% are 30–44 years old [1]. Advances in tumor diagnosis and treatment technologies have significantly improved the average life expectancy of patients as well as the 5-year survival rate of female patients with cancer in China [2]. However, common chemotherapy drugs, such as cyclophosphamide (CTX), cause damage to oocytes and granulosa cells (GCs) [3], leading to a loss of fertility and reproductive endocrine function, thereby increasing the risk of premature ovarian failure (POF) in treated women [4].

Mesenchymal stem cells (MSCs) have differentiation potential and are involved in homing, tissue cell regeneration, immune regulation, and the repair of damage in certain diseases [5, 6]. Studies aimed at the development of POF treatments have shown that the transplantation of MSCs can inhibit apoptosis in ovarian GCs and improve follicular development at various stages [7–9]. Knockout studies using rats have confirmed that nerve growth factor (NGF) is an important factor for primordial follicular growth in the nongonadotropin-dependent phase. NGF affects follicle survival in a dose-dependent manner* in vitro* and can induce FSHR expression [10]. In this study, human umbilical cord mesenchymal stem cells (UCMSCs) were transplanted by tail vein injection, and the expression levels of NGF and its receptor TrkA were evaluated in ovarian tissues during the repair process in POF rat models. This study provides insight into the potential therapeutic efficacy of UCMSCs for POF as well as the underlying mechanism.

## 2. Materials and Methods

### 2.1. Animals

A total of 40 specific pathogen-free (SPF) female Sprague–Dawley (SD) rats (age, 12 weeks; no birth; average weight, 216 ± 12 g) were provided by the Zhejiang Provincial Animal Experimental Center. The rats were housed with a previous method [11]. This project was approved by the Medical Ethics Committee of Jinhua Polytechnic (Jinhua, China).

### 2.2. Cell Culture and Identification

UCMSCs were provided by Jinhua Sidanmu Stem Cell Biotechnology Co., Ltd. (Jinhua, Zhejiang, China). UCMSC surface marker expression was analyzed at passage three by flow cytometry, using fluorescein isothiocyanate (FITC) conjugated monoclonal anti-human CD90 and CD45 antibodies, phycoerythrin (PE)-conjugated anti-human CD34, CD73, and CD105 antibodies, and allophycocyanin (APC)-conjugated anti-human CD19 and CD14 antibodies. For the flow cytometry analysis, adherent cells were detached by treatment with 0.25% trypsin-EDTA (Thermo Fisher Scientific, Inc., Waltham, MA, USA), neutralized with culture medium containing FBS, and disaggregated into single cells by pipetting. The cells were incubated with mAbs for 30 min at 4°C, washed twice with PBS, resuspended in 0.5 mL of PBS, and immediately analyzed using a NovoCyte Flow Cytometer (ACEA Biosciences, Inc., San Diego, CA, USA). At least 2 × 10^5^ cells were used for each sample and Cell Quest software was used for data analysis.

### 2.3. POF Model

**Figure 1:**
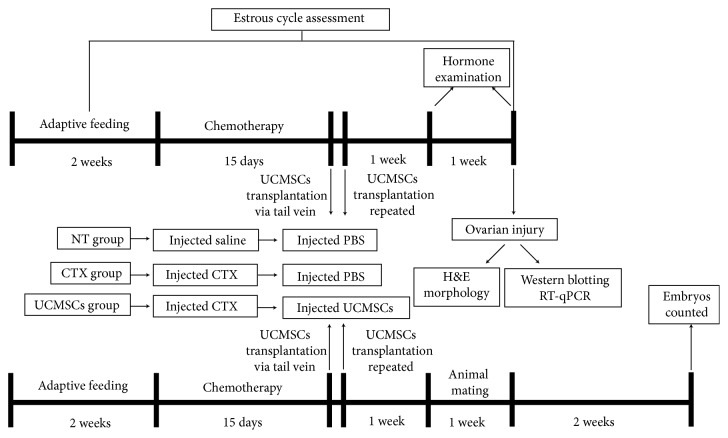
Schematic of the experimental procedure used to explore the effects of UCMSCs transplantation on chemotherapy-induced ovarian failure in rats.

After 2 weeks of adaptive feeding, rats were divided into two groups, the NT group (12 rats) and the experimental group (28 rats). In the experimental group, the rats received an intraperitoneal injection of cyclophosphamide (CTX) as previous description [11]. Then, the experimental rats were randomly divided into two groups of 12 rats each, namely, the CTX group and the UCMSC group. The process is shown in Figure 1.

### 2.4. Cell Transplantation

Rats in the UCMSC group were injected intravenously with 5 × 10^6^ UCMSCs in 500 *μ*L of PBS according to previously described methods [7, 8]. Rats in the NT and CTX groups were injected with 500 *μ*L of PBS alone. The injections were repeated once on the following day.

### 2.5. Rat Estrous Cycle Assessment

Following adaptive feeding for approximately 7 days, the estrous cycles of the rats were monitored continuously by vaginal smear referring to our previous method [11]. And the estrous cycle stages, including proestrus, estrus, metestrus, and diestrus, were determined according to a previous description [12].

### 2.6. Hormone Examination

Rats were anesthetized with pentobarbital (5 mg/100 g, intraperitoneal injection) the day before cell transplantation and 1 week and 2 weeks after transplantation. Approximately 1 mL of blood was withdrawn for hormone examination including anti-Mullerian hormone (AMH), estradiol (E2), follicle-stimulating hormone (FSH), and gonadotropin releasing hormone (GnRH) by using ELISA Kits (Cusabio Technology, LLC, Wuhan, China) according to our previous description [11].

### 2.7. Hematoxylin and Eosin Staining of Ovary Slices

The ovaries of three groups of rats were collected 2 weeks after transplantation, and bilateral ovaries were collected for subsequent experiments. According to previously described methods [11, 13], the ovarian tissue was stained with hematoxylin and eosin, and the follicles were detected and classified as primordial, primary, secondary, and early antral follicles.

### 2.8. Western Blotting

Ovarian tissues were placed in cold RIPA lysis buffer (Wuhan Boster Biological Technology, Ltd., Wuhan, China). Tissue blocks were homogenized on ice for 15 min and centrifuged at 14,000 ×* g* for 30 min at 4°C. The protein concentration in each sample was determined using a BCA Protein Quantification Kit (Wuhan Boster Biological Technology, Ltd.). Equal amounts of protein were boiled with 5× loading buffer for 5 min and loaded onto a 12% sodium dodecyl sulfate-polyacrylamide gel. Electrophoresis was performed at 60 V for 30 min and 80 V for 120 min. Separated proteins were transferred onto a polyvinylidene difluoride membrane at 100 V for 120 min. Membranes were blocked with 5% dried skim milk (Wuhan Boster Biological Technology, Ltd.) overnight at 4°C and incubated with the following primary antibodies: rabbit polyclonal anti-NGF (1:1000), rabbit polyclonal anti-FSHR (1:500), rabbit polyclonal anti-TrkA (1:500), and rabbit polyclonal anti-caspase-3 (1:500; all Abcam, Cambridge, UK) overnight at 4°C. Equal loading of protein samples was confirmed by subsequent Tubulin immunoblots (1:1000; Abcam). Immunodetection was performed using SuperSignal™ West Dura Extended Duration substrate (Thermo Fisher Scientific, Inc.) following incubation with the goat anti-rabbit IgG secondary antibody (1:5000; Thermo Fisher Scientific, Inc.) for 1 h at 37°C. The X-ray films were developed, images were captured, and Quantity One (Bio-Rad, Hercules CA, USA) was used to analyze grayscale values.

### 2.9. Reverse Transcription-Quantitative Polymerase Chain Reaction (RT-qPCR)

The ovarian tissues were ground in liquid nitrogen. Total RNA was extracted using TRIzol reagent (Invitrogen; Thermo Fisher Scientific, Inc.). The purity and concentration of RNA were determined by spectrophotometry. Subsequently, total RNA (1 *μ*g) was reverse-transcribed into cDNA using the iScript™ cDNA Synthesis Kit (Bio-Rad Laboratories, Inc.). Specific primers were designed using Primer Premier 6 and synthesized by Sangon Biotech Co., Ltd. (Shanghai, China). The primer sequences are listed in Table 1. ITaq Universal SYBR Green Supermix (Bio-Rad Laboratories, Inc.) was used for qPCR with a Bio-Rad CFX96 detection system (Bio-Rad Laboratories, Inc.). The following thermocycling conditions were used: initial denaturation at 95°C for 60 s, followed by 40 cycles of 95°C for 15 s and 60°C for 40 s. The Ct value for each sample was subtracted from the value of the internal control gene to generate ΔCt. The 2^−ΔΔct^ values were subsequently analyzed.

### 2.10. Assessment of Natural Fertility

After 1 week of adaptive feeding, 30 female rats (age, 12 weeks; no birth; average weight, 214 ± 4 g) were divided into 3 groups (NT group, CTX group, and UCMSC group) to construct POF models and transplanted with UCMSCs as mentioned above. After one week, animal mating was initiated by cohabitating them with males at a ratio of 2 females: 1 male. Animals were cohabited at 5:00 p.m., and vaginal smears were taken at around 9:00 a.m. on the second day to ensure fruitful mating for 1 week. Animals that mated successfully were no longer cohabited. Two weeks later, rats were anesthetized with pentobarbital (5 mg/100 g, intraperitoneal injection) and sacrificed by cervical dislocation. The uterus was taken out, and the number of embryos was counted. The process is shown in Figure 1.

### 2.11. Statistical Analysis

Data are expressed as means ± SEM and were analyzed using SPSS 16.0 (IBM, Armonk, NY, USA). One-way analysis of variance followed by Tukey's post hoc test was used to determine statistical significance. The chi-squared test was used for comparison between groups.* P* < 0.05 indicated statistical significance.

## 3. Results

### 3.1. Isolation and Characterization of Human UCMSCs

**Figure 2:**
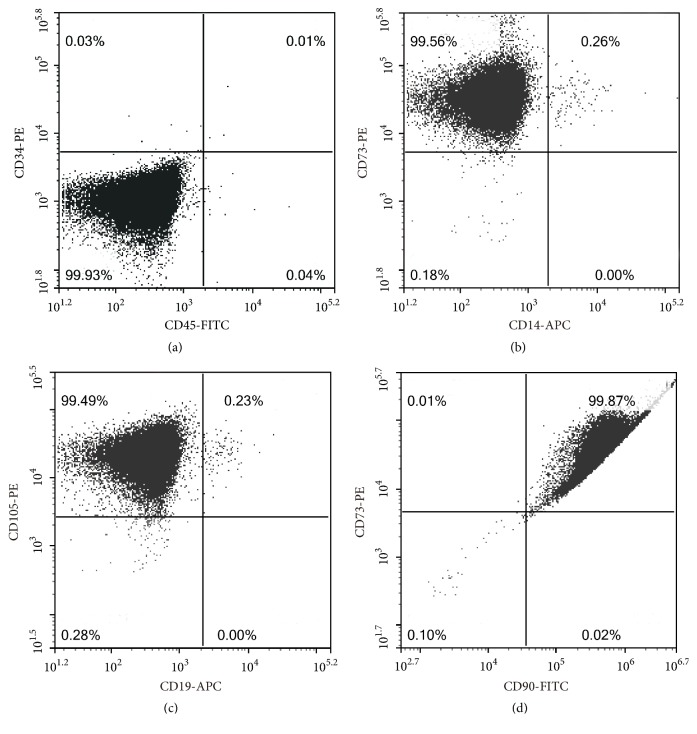
Identification of mesenchymal stem cells by flow cytometry. (a) CD34 (PE) and CD45 (FITC) were negative; (b) CD14 (APC) was negative and CD73 (PE) was positive; (c) CD19 (APC) was negative and CD105 (PE) was positive; (d) CD90 (FITC) was positive.

UCMSC surface marker expression was analyzed using cells at passage three by flow cytometry. Low expression levels of CD34, CD45, CD14, and CD19 (Figures 2(a), 2(b), and 2(c)) and high levels of CD73, CD105, and CD90 were detected (Figures 2(b), 2(c), and 2(d)).

### 3.2. UCMSC Transplantation Improved Hormone Secretion in POF Rats

**Figure 3:**
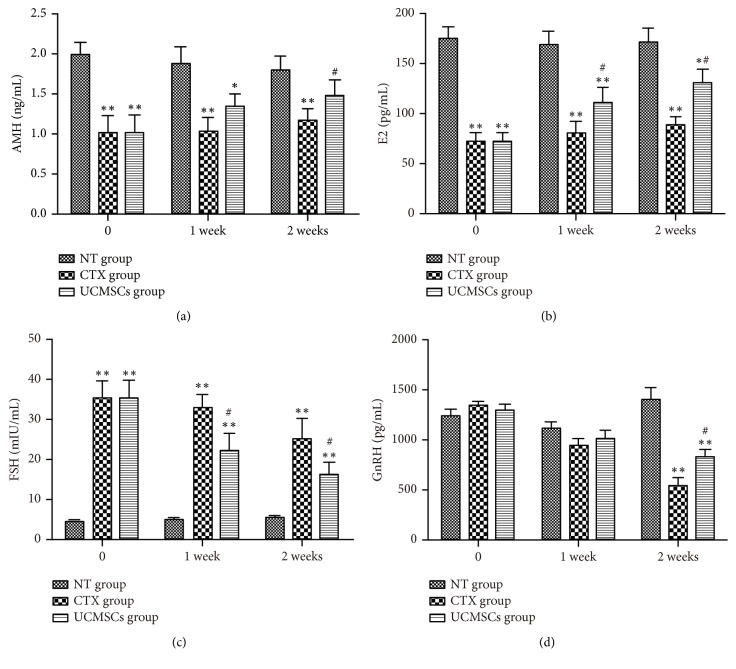
UCMSCs transplantation effect on the serum levels of E2, AMH, FSH, and GnRH in rats. (a) is the serum levels of AMH in three groups; (b) is the serum levels of E2 in three groups; (c) is the serum levels of FSH in three groups; (d) is the serum levels of GnRH in three groups. Data are presented as the mean ± SEM, n = 12/group. *∗P*<0.05; *∗∗P*<0.01, compared with NT group; #*P*<0.05; ##*P*<0.01 compared with CTX group.

Blood was obtained from rats in each group before cell transplantation and 1 week and 2 weeks after transplantation. Before cell transplantation, the levels of E2 and AMH in the CTX group and UCMSC group were lower than those in the NT group. However, FSH levels were higher than those in the NT group. The E2 and AMH levels 1 week after transplantation in the UCMSC group were significantly higher than those in the CTX group (*P *< 0.05), while FSH levels were significantly lower (*P *< 0.01). In addition, the levels of E2, AMH, and GnRH at 2 weeks after transplantation in the UCMSC group were significantly higher than those in the CTX group (*P *< 0.05), while FSH levels were significantly lower (Figure 3).

### 3.3. UCMSC Transplantation Prolonged Estrus in POF Rats

Before the establishment of the CTX model, all rats in the three groups presented regular estrous (4.58 ± 0.59) and diestrus stages (3.18 ± 0.61). At the end of the study period, rats injected with CTX showed shortened estrous (2.64 ± 0.59) and prolonged diestrus (4.63 ± 0.59). Two weeks after UCMSC transplantation, estrous in the UCMSC group (4.12 ± 0.42) was prolonged compared with that in the CTX group (3.21 ± 0.56,* P* < 0.05).

### 3.4. UCMSC Transplantation Decreased Caspase-3 Expression in the Ovarian Tissues of POF Rats

**Figure 4:**
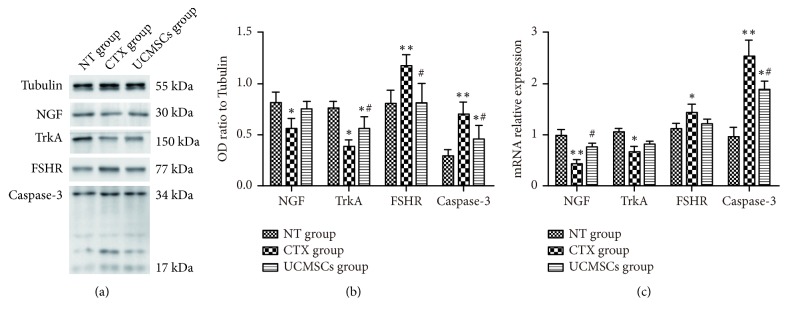
UCMSCs transplantation effect expression of NGF, TrkA, FSHR, and caspase-3 in the ovarian tissues of rats. (a) Bands for the three groups are shown, molecular weight of NGF is 30 kDa, FSHR is 77 kDa, TrkA is 150 kDa, Tubulin is 55 kDa, and caspase-3 is 34 and 17 kDa; (b) the gray value ratio of each protein to GAPDH in the three groups is shown; (c) mRNA relative expression of NGF, TrkA, FSHR, and caspase-3 detected by RT-qPCR is shown. *∗P*<0.05; *∗∗P*<0.01, compared with NT group; #*P*<0.05; ##*P*<0.01 compared with CTX group.

To evaluate apoptosis in ovarian tissues, caspase-3 expression was detected by western blotting and RT-qPCR. Two weeks after transplantation, the mRNA expression levels of caspase-3 were significantly lower in the ovarian tissues of the UCMSC group than in those of the CTX group (*P* < 0.05, Figure 4). Protein expression levels of caspase-3 were significantly lower in the ovarian tissues of the UCMSC group than in those of the CTX group (*P* < 0.05, Figure 4).

### 3.5. UCMSC Transplantation Prevents the Loss of Secondary Follicles

**Figure 5:**
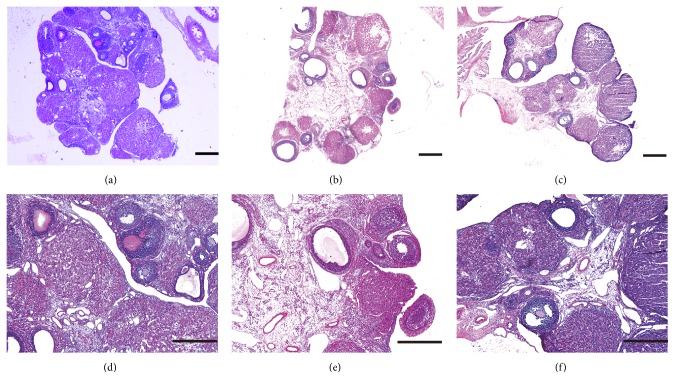
Morphological alterations in the ovarian tissues of rats after UCMSCs transplantation. Tissues are stained by hematoxylin-eosin. In NT group, ovarian structure was normal, and there were large amounts of primordial follicles and a well-developed corpus luteum (a, d). In CTX group, ovarian stromal suffered structural disorder, severe fibrosis, and cortical thickening, the corpus luteum exhibited fibrosis, and the number of follicles at each stage reduced (b, e). In UCMSCs group, follicle at different stages could be seen (c, f). Scale bar=200 *μ*m.

The primordial follicles in the three groups were evaluated by HE staining, and representative images are shown in Figure 5. The numbers of follicles at each stage in the three groups are shown in Table 2. There were significantly more secondary follicles in the UCMSC group than in the CTX group 2 weeks after transplantation (*P* < 0.05).

### 3.6. UCMSC Transplantation Increased TrkA Expression and Decreased FSHR Expression in Ovarian Tissues of POF Rats

Two weeks after transplantation, the mRNA expression level of NGF was significantly higher in the ovarian tissues of the UCMSC group than the CTX group (*P* < 0.05; Figure 4). Protein expression level of TrkA were significantly higher in the ovarian tissues of the UCMSC group than the CTX group, while expression level of FSHR was decreased (*P* < 0.05; Figure 4).

### 3.7. UCMSC Transplantation Improved Pregnant Rate and Embryos Numbers of POF Rats

Rats in NT group were all pregnant, having a conception rate of 100%. The number of embryos was 11.7 ± 1.49 per mother rat. The conception rate in the CTX group was 40%, the number of embryos was 9.5 ± 1.29 per mother rat. The conception rate in the UCMSC group was 60%, and the number of embryos was 10.5 ± 1.05 per mother rat. The latter two groups were statistically different from the NT group (*P *< 0.05), and the UCMSC group was statistically different from the CTX group (*P *< 0.05). The results are shown in Table 3.

## 4. Discussion

The endocrine system produces a variety of steroid hormones that regulate the growth and survival of follicles by paracrine or autocrine mechanisms. Elevated levels of FSH accelerate the depletion of follicle storage and result in decreased ovarian reserves [14, 15]. E2 primarily produced by GCs has negative feedback effects on FSH in the hypothalamic-pituitary-ovarian axis. GCs are the most important stromal cells in the ovary; they surround the oocyte and play a key role in folliculogenesis [16]. Excessive apoptosis in GCs is induced by chemotherapeutic agents, resulting in a decrease in E2 levels [17]. The interruption of this negative feedback results in an uncontrolled increase in FSH levels [18]. Chemotherapy causes interstitial fibrosis of ovarian tissues, and follicles at all stages are reduced, especially antral and secondary follicles. Some researchers believe that this phenomenon can be attributed to increased GC proliferation [8].

Stem cell transplantation is a potential treatment for damaged tissues [19] and a powerful tool for restoring fertility and pregnancy [7]. Various types of stem cells [7, 20, 21] have recently been studied for the treatment of POF; these studies have shown that stem cell transplantation is an ideal treatment for POF. UCMSCs have several advantages over other stem cells, such as poor immunogenic properties, easy isolation, expansion* in vitro*, and minimal ethical issues [22]. In our study, UCMSCs were positive for the cell surface markers CD73, CD90, and CD105 and negative for CD14, CD19, CD34, and CD45. There was a significant reduction in cell apoptosis after UCMSC transplantation and an improvement in folliculogenesis in the POF rat models. Two weeks after transplantation, ovarian function improved, as evidenced by the restoration of the estrous cycle, increased E2 levels and follicles, and decreased FSH levels. Transplanted MSCs are located in the interstitium and are not found in follicles, indicating that they may play an important supportive role in the ovarian microenvironment and would not differentiate into oocytes or GCs [8, 23].

The TrkA receptor predominantly activates phosphatidylinositol-3-kinase (PI3K) and mitogen-activated protein kinase (MAPK) to promote cell survival and proliferation [24]. In both oocytes and GCs, a complete PI3K/Akt signaling system coordinates and regulates follicular growth, maturation, and periodic ovulation [25]. The PI3K/Akt signaling pathway regulates oocyte growth and the survival and development of primordial follicles, promotes the proliferation and differentiation of GCs, and inhibits apoptosis, and these processes are critical for the normal development and physiological functions of the ovaries [26, 27]. Two weeks after UCMSC transplantation, the expression of NGF and TrkA increased and caspase-3 decreased in the ovary, supporting the notion that the protective effect of transplanted UCMSCs is mediated by the secretion of NGF to prevent GC apoptosis. MSCs could secrete a number of other angiogenic growth factors, such as hepatocyte growth factor (HGF), vascular endothelial growth factor (VEGF), placental growth factor (PGF), and TGF-*β*. These factors are involved in the repair of damaged ovarian function [21, 28]. Whether NGF acts synergistically with these factors should be examined in further research.

The intravenous injection of UCMSCs could partially ameliorate changes in hormonal function, folliculogenesis, and architecture in POF rats caused by CTX. Based on their elevated levels, NGF and TrkA might mediate the effect of UCMSCs on the restoration of ovarian function. The fertility of the rat model was improved by UCMSC transplantation in this study. However, the tumorigenicity of these cells could not be excluded, and whether the pups would have abnormalities, mutations, etc., was not assessed. Further, deeper investigations are required to better understand the exact underlying mechanism and the safety of the therapeutic effects of UCMSCs on POF.

## 5. Conclusions

UCMSCs could reduce ovarian failure due to premature senescence caused by chemotherapy, and the NGF/TrkA signaling pathway was involved in the amelioration of POF.

## Figures and Tables

**Table 1:** Specific primer sequences designed by Primer Premier 6.

Gene	Primer Sequence(5′-3′)	Length (bp)
Rat* FSHR*	TGACCACAAGCCAATACAA	489
	TATAGCAGCCACAGATGAC	
Rat *NGF*	TCTTCGGACACTCTGGATT	162
	CGTGGCTGTGGTCTTATC	
Rat *TrkA*	ACTAACAGCACATCAAGAGA	407
	TCATTCAGAAGGTTGTAGCA	
Rat *GAPDH*	TTCAACGGCACAGTCAAG	116
	TACTCAGCACCAGCATCA	
Rat *Caspase-3*	ACTTGGTTGGCTTGTTGA	212
	GTATTATGGTCTGTTCCTGTAG	

**Table 2:** The follicle number increased after UCMSCs transplantation.

Group	Primordial	Primary	Secondary	Early antral
NT group	112.4±34.55	23.8±4.52	19.6±5.34	11.7±3.18
CTX group	63.8±22.72*∗*	14.1±3.89*∗∗*	8.4±2.81*∗∗*	2.3±1.72*∗∗*
UCMSCs group	70.1±18.14*∗*	17.4±2.24	14.5±3.34#	4.4±1.98*∗∗*

*∗P*<0.05 and *∗∗P*<0.01, compared with NT group; #*P*<0.05, compared with CTX group.

**Table 3:** Comparison of pregnancy rate and embryos numbers among groups.

Group	Pregnancy rate	Embryos numbers
NT group	100	11.7±1.49
CTX group	40*∗∗*	9.5±1.29*∗*
UCMSCs group	60*∗∗*#	10.5±1.05

*∗P*<0.05 and *∗∗P*<0.01, compared with NT group; #*P*<0.05, compared with CTX group.

## Data Availability

The data used to support the findings of this study are available from the corresponding author upon request.
